# Acceptance of Pharmaceutical Services by Home-Dwelling Older Patients: A Case Study in a Portuguese Community Pharmacy

**DOI:** 10.3390/ijerph18147401

**Published:** 2021-07-11

**Authors:** Ana Rita Paiva, Ana Isabel Plácido, Isabel Curto, Manuel Morgado, Maria Teresa Herdeiro, Fátima Roque

**Affiliations:** 1 Health Sciences School, Polytechnic of Guarda, Rua da Cadeia, 6300-035 Guarda, Portugal; anaritapaiva970@gmail.com (A.R.P.); anaplacido@ipg.pt (A.I.P.); mmorgado@ipg.pt (M.M.); 2 Research Unit for Inland Development, Polytechnic of Guarda (UDI-IPG), Av. Dr. Francisco Sá Carneiro 50, 6300-559 Guarda, Portugal; 3 Pharmacy Mousaco Torrão, Estrada Municipal 506 11, R/C, 6200-571 Ferro, Portugal; isabelmcurto@gmail.com; 4 Health Sciences Research Centre, University of Beira Interior (CICS-UBI), 6200-506 Covilhã, Portugal; 5 Pharmaceutical Services of University Hospital Centre of Cova da Beira, 6200-251 Covilhã, Portugal; 6 Department of Medical Sciences, Institute of Biomedicine, University of Aveiro (iBIMED-UA), 3810-193 Aveiro, Portugal; teresaherdeiro@ua.pt

**Keywords:** community pharmacy, older adults, polypharmacy, medication adherence

## Abstract

Background: Aging-related comorbidities predispose older adults to polypharmacy and consequently an increased risk of adverse drug reactions and poor compliance. Pharmacists’ interventions can have a beneficial impact on the improvement of clinical outcomes. Thus, this work aimed to assess the acceptance of Portuguese home-dwelling older adults regarding a pharmaceutical service paid by patients for medication management and pharmacotherapy follow-up. We also intended to analyze medication, characterize the medication consumption profile, and identify the main difficulties of our sample during their daily medication management. Methods: A questionnaire on adherence and medication therapy management was applied to polymedicated patients ≥65 years old, in a community pharmacy. Results: Of the 88 participants, 92.2% would be willing to pay for a pharmacotherapy management service, and 75.6% answered that they would be willing to pay for an individual medication preparation service. In addition, 45.7% of the participants were categorized as lower adherents to a medication therapeutic regimen. Our sample reported that during their daily lives, they felt difficulty: to remember to take their pills (17%), to manage so many medicines (15.9%), and to swallow the pills (9.1%). Conclusions: Polymedicated older adults are willing to pay for a service to improve the management of their medicines, suggesting that they recognize the role of pharmacists in medication management. This study provides useful information for the conceptualization of a pharmacotherapy management service that includes medication review and a pharmacotherapy follow-up.

## 1. Introduction

The world population is aging, increasing the consumption of medicines and the occurrence of polypharmacy [1]. Polypharmacy, the consumption of five or more medicines [2], is associated with the loss of resilience and decreased cognitive abilities due to aging, potentiating the occurrence of drug-related problems (DRPs) and resulting in poor compliance and failure of treatment and leading to an annual increase in worldwide expenditure of approximately USD 42 billion and creating a need for interventions to improve pharmacotherapy [3,4,5].

A more active role of pharmacists in medication management could have positive impacts on clinical outcomes [6,7]. Indeed, pharmaceutical interventions can reduce the number of DRPs [5,7]. However, according to the literature, the beneficial effects of a medication therapy management pharmaceutical service can only be achieved if the analysis of pharmacotherapy is systematically carried out [8].

Portugal is one of the most aged countries in the world, and the inner central region is the region with the second highest aging index in the country [9,10]. A recent study reports that 83.0% of the Portuguese population that reside in the inner central region of Portugal are responsible for their own medicine management [11]. Moreover, 25.0% of them did not have any educational degree and almost 60.0% of them had monthly incomes of less than EUR 439 [11]. A previous study of our group revealed that older adults valorize their medicines and, for this reason, they frequently develop strategies to improve medication adherence [12]. However, due to the low health literacy, their efforts do not have the expected beneficial results, resulting in a lack of therapeutic effectiveness [12,13]. In this context, we developed this work intending to assess the acceptance of Portuguese home-dwelling older adults regarding a pharmacist patients-pay service for medication management and pharmacotherapy follow-up. This work also aimed to analyze medication adherence in Portuguese home-dwelling older adults as well as to identify the behaviors and beliefs of older adults that can influence compliance with the medication therapeutic regimen.

## 2. Materials and Methods

### 2.1. Ethics Approval and Consent to Participate

This study obtained the ethical approval of the ethical committee of the Polytechnic Institute of Guarda (registry no. 8/2019), and authorization was obtained from the pharmacist–technical director of the community pharmacy where the survey took place. A written informed consent was obtained from all participants before inclusion in the study.

### 2.2. Study Design and Data Collection

A cross-sectional study was conducted with a convenience sample in a community pharmacy located in the inner central region of Portugal between December 2019 and February 2020. Participants were selected based on their clinical and pharmacotherapeutic profile, recorded in the community pharmacy computer database. The selected population included home-dwelling patients 65 years or older taking five or more medicines. Patients with visible difficulties that prevented them from responding appropriately were excluded from the study. The questionnaire was applied by an interviewer and was divided into five sections, the first section containing multiple-choice questions about daily administration of medications; the second and third sections were adapted from a previously validated questionnaire for the Portuguese population, containing the evaluation of therapeutic adherence with the “Medida de Adesão aos tratamentos” (MAT) scale, by Delgado et al. [14], validated for the Portuguese population [11]. The MAT scale consists of 7 items rated on a six-point Likert scale ranging from “Always” to “Never”, and in all questions “Always” represents the lowest adherence and “Never” is the highest.

The fourth section contained questions related to the opinion of older adults regarding a pharmacist patients-pay service for medication management and pharmacotherapy follow-up; in the fifth section, to evaluate the degree of knowledge as well as the daily management of each medicine, patients were invited to observe drug packaging, one at a time, and answer to questions regarding the daily management and the concerns regarding each medicine.

The list of medicines was converted to the corresponding Anatomical Therapeutic Classification (ATC) code, using the WHO Collaborating Centre for Drug Statistics Methodology website [15].

### 2.3. Statistical Analysis

Data analysis was executed using the Statistical Package for Social Sciences (SPSS 25, IBM Corp., New York, NY, USA). Numerical and ordinal data were analyzed using descriptive statistics and presented as frequency and percentage and the mean, median, and standard deviation. The “do not know/did not answer” answers were addressed and considered as missing values. The medication adherence level from each individual was obtained by summing up the values from the seven questions and dividing the value by the number of questions. The classification as adherent and nonadherent used the median as the cutoff value. Below the median, the sample is nonadherent and, above it, is adherent. To clear the results, whenever the adherence classification was involved, the sample within the median value was excluded [11].

## 3. Results

### 3.1. Sample Characterization

Table 1 presents the sociodemographic characteristics of the sample. Of the 106 invited participants, 16 refused to participate and two were excluded because, at the time of the interview, they were taking fewer than five medicines.

The median (Q1–Q3) of prescribed medicines was 7.0 (6.0–8.0). About 43.10% of the consumed medicines were generic (Table 1) and it was observed that 7.95% of the participants consumed food supplements daily.

### 3.2. Daily Medication Management

Although all participants were responsible for managing their medicines, 65.90% of them did not know the names of their medicines. A total of 70.50% of participants said that they do not feel any difficulty during their daily medication routine, while 17.00% admitted that it was hard to remember the correct way to take their medicines and to manage the schedules (Table 2).

Finally, 84.10% of the participants affirmed that when their general practitioners prescribe a new medicine, they do not ask for information about the new medicine, and admitted that they only sometimes ask for more information from the community pharmacists (8.00%) or the general practitioner (5.60%).

### 3.3. Medication Adherence

After the analysis of the MAT scale (Table 3), it was observed that 46.70% of the sample had an adherence value below the median (5.57) (Table 4) and were categorized as lower adherents.

### 3.4. Medication Consumption Profile

A total of 640 medicines were prescribed to patients. Within the fourth level of ATC, the chemical/therapeutic/pharmacological subgroups A02BC “Proton pump inhibitors”, C10AA “HMG CoA reductase inhibitors”, and N05BA “Benzodiazepine derivatives” were the most frequently consumed medicines and were consumed by 62.2%, 50.0, and 36.6%, respectively, of the sample (Table 1). The routes of administration most frequently used by the participants were oral and inhalation used, respectively, by 100% and 14.4% of the sample. Of the 591 drugs administered orally, the most frequent dosage forms used by the participants were tablets (520, consumed by 100% of the participants) and capsules (71, consumed by 54.4% of the participants). Of the 512 tablets consumed by the participants, 50 tablets were modified-release tablets and were consumed by 38 participants (43.2%) (supplementary material, Table S1).

### 3.5. Patient-Related Problems

When asked about each medicine (in the presence of the drug packaging), 66.3% of the patients were able to identify the therapeutic indication of more than 75% of their medicines, while 10.9% of the patients identified less than 50% of their medicines. When asked how long they had been taking that specific medicine, the majority affirmed that they do not remember. According to the answers given by the patients, the majority of the medicines (76.3%) were taken once a day, 16.7% were taken two times daily, 1.9% were taken more than two times daily, and the remaining pills were taken once weekly or more. About 59.5% of the medicines were taken early in the morning, when fasting or at breakfast, 11.8% during lunch, 2.1% were taken during the afternoon, 26.7% were taken at dinner, and 14.1% before sleeping. When specifically asked how they took each medicine, it was observed that all patients take at least one medicine during the meal, and 6.7% of the participants affirmed that they do not remember how they take at least one of their tablets. When directly asked about the beverage that they pick to take each tablet, it was observed that 96.7% of the participants take at least one pill with water and 2.2% of the participants take at least one tablet with coffee. Of the 40% of the participants that affirmed that they split their tablets, 4.3% of them split modified-released tablets (Table 5).

When asked whether they have ever reduced or not taken each specific medicine, 21.7% of the older participants answered affirmatively. The main reason for this behavior is forgetfulness (11.9%), dislike of taking the medicine (7.6%), and the cost of the medicine (2.2%). In the presence of the drug packaging, participants had the opportunity and freedom to express their opinions and concerns regarding their medicines, and 5.5% of them reported experiencing adverse events (ADR) that have been previously documented. The medicines reported as responsible for ADR were torasemide, risperidone, valsartan + amlodipine, esomeprazole, and amlodipine + olmesartan medoxomil + hydrochlorothiazide.

### 3.6. Older Patients’ Opinions Regarding Community Pharmacist Role in Pharmacotherapeutic Management

When questioned if they would like to have help in their daily management of medicines, only 6.8% answered positively, and the remaining 93.2% affirmed that they are capable of managing their medicines without help.

However, surprisingly, 92.2% of the patients affirmed that they would be willing to pay for a pharmacotherapy service and 77.3% affirmed that they would be willing to pay for an individual medication preparation service.

The main reasons presented for the rejection of the pharmacotherapy service were the fact that the participants believed that this type of service should be performed by their general practitioner. The 22.7% of the participants that rejected the medication preparation service gave the following main reasons: (a) they do not like this type of service (11.4%), (b) they preferred to take the medicines from the original box (5.7%), or (c) they believe that this type of service is irrelevant (3.4%).

## 4. Discussion

In this study, we found that our sample of older adults from a community pharmacy in the inner central region of Portugal recognized the importance of pharmaceutical services and are willing to pay for a pharmacotherapy management service and an individual medication preparation service.

As older adults initially answered that they are capable of managing their medicines alone, we believed that they would be willing to pay for a pharmacotherapy service due to their perception that their health status is not good and that pharmacists can provide valuable collaboration in optimizing drug therapy rather than the recognition that their non-compliance to treatments can compromise the therapeutic outcomes.

The valuable work of pharmacists on polypharmacy reduction and in the improvement of prescription quality and the impact of this medication management on health outcomes cannot be refuted [16,17,18,19]. Pharmacists can have an important role in the reduction of medication errors, poor adherence, and hospitalization [20,21]. The strength of patient–pharmacist interactions allows the pharmacists to obtain relevant information regarding the health outcomes of older adults. The assessment of this information by the physician can not only be helpful in the management of polymedicated older adults but can also lead to a reduction in health care costs [22].

During this study, it was observed that the participants have different behaviors, i.e., in the first part of the study, they tended to answer the questions regarding their medicines in general, and answered according to what they believe is the correct way to manage their medicines. However, in the second part of the study, the presence of their medicines’ packaging led the patients to give more specific and assertive answers, which facilitated the identification of potential DRPs. The main factors associated with these DRPs were forgetfulness, the cost of medicines, and the disfavor of some pills/pathologies by patients [11]. Previous studies have also pointed out that economic factors and the lack of knowledge of patients are preponderant factors in the lack of therapeutic adherence [3,11,23,24]. Some participants also pointed out the fear of an adverse reaction as a relevant factor in poor compliance. This observation is in concordance with other studies that reported that patients fear experiencing adverse reactions that they have read about in the package leaflet of some medicines or that someone has told them that may occur or even that they believe may occur due to chronic drug use [25,26]. In clinical practice, the pharmacotherapeutic monitoring of patients can facilitate the detection of DRPs related to drug selection, dose selection, or treatment duration [27,28]. We believe that due to the close relationship with patients, pharmacists can play a key role in improving the user’s clinical outcomes, by promoting correct practices in the use of medicines and simplifying the therapeutic regimen [29,30,31,32]. Considering that less than 50% of the medicines consumed are generic, we also believe that pharmacists can also have an important role in counseling patients regarding the use of generic medicines that are cheaper and equally safe.

This study reinforces data from another previous study of our research group [11], suggesting that older adults are poor adherents and committed a high number of medication errors. This study also suggests that older adults valorize the role of community pharmacists and are able to accept the help of the community pharmacist to improve their medication management and consequently the promotion of medication adherence and decrease in medication errors.

However, although this study provides relevant data regarding the opinions of older adults regarding medication management, this work had some limitations related to the limited number of participants and the convenience sampling and cannot be generalized to all Portuguese populations. In an increasingly aging world, these data are valuable to understanding the needs of older adults regarding the management of medicines.

## 5. Conclusions

Although the majority of older adults believed they can manage their medicines without help, more than 30% of them experienced at least one patient-related DRP. Moreover, the majority of them recognized the role of pharmacists in the achievement of better health outcomes, and they were willing to pay for a pharmacotherapy management service.

Despite the small sample, this study provides valuable information for the implementation of a pharmacotherapy management service directed at older adults. The patient-related problems observed also suggested that educational strategies directed at older adults should be considered to minimize medication errors.

## Figures and Tables

**Table 1:** Sociodemographic characteristics of the sample.

n = 88				n (%)
Female				63 (71.6)
Age: median (Q1—Q3): 74.0 (69.0–80.8)				
Monthly income				
EUR ≤ 439				39 (44.3)
EUR 440–580				30 (34.1)
EUR 581–1160				8 (20.5)
EUR ≥ 1161				1 (1.1)
Whom do you live with?				
Alone				37 (42.0)
Partner				46 (52.3)
Others				5 (5.7)
Education level				
Does not read or write				9 (10.2)
Knows how to read/write but no grade				4 (4.5)
Primary school				63 (71.6)
2nd cycle (5th and 6th grade)				5 (5.7)
3rd cycle (7th to 9th)				2 (2.3)
High school (10th to 12th)				2 (2.3)
Higher education/graduate				3 (3.4)
Prescribed medicines, median (Q1–Q3): 7.0 (6.0–8.0)				
5–9				76 (86.4)
≥10				12 (13.6)
Generic medicines, median (Q1–Q3): 3.0 (2.0–4.0)				
1–3				41 (46.5)
4–6				38 (43.3)
7–10				6 (6.8)
≤11				2 (2.3)
Brand-name medicines, median (Q1–Q3): 4.0 (3.0–5.0)				
1–3				41 (46.5)
4–6				38 (43.3)
7–10				6 (6.8)
≤11				2 (2.3)
Most prescribed medicines *n* (%)				
A: Alimentary tract and metabolism				76 (86.4)
	A02: Drugs for acid-related disorders			57 (64.8)
		A02B: Drugs for peptic ulcer and gastroesophageal reflux disease (GORD)		57 (64.8)
			A02BC: Proton pump inhibitors	55 (62.5)
C: Cardiovascular system				86 (96.6)
	C10: Lipid modifying agents			54 (61.1)
		C10A: Lipid modifying agents, plain		51 (68.0)
			C10AA: HMG CoA reductase inhibitors	43 (48.9)
N: Nervous system				58 (65.9)
	N05: Psycholeptics			32 (36.4)
		N05B: Anxiolytics		31 (35.2)
			N05BA: Benzodiazepine derivatives	31 (35.2)

**Table 2:** Main difficulties experienced by participants with medicines and main reasons for not complying with the therapeutic regimen.

Identification of Medicines *n* (%)	
Name	30 (34.1%)
Color of the pills/capsules	13 (14.8%)
Box/packaging	68 (77.3%)
Shape of the pills	17 (19.3%)
**Main Difficulties in Their Daily Life Management of Medicines *n* (%)**	
Forgetting to take medication	15 (17.0%)
Management of the schedule of so many medicines	14 (15.9%)
Opening the box/blister/flasks	4 (4.5%)
Swallowing	8 (9.1%)
None	62 (70.5%)
**The Most Frequent Strategies Used by the Participants to Avoid Forgetfulness *n* (%)**	
Medication box	22 (25.0%)
Put the pills in different bags/places according to the schedule in medicines must be taken	12 (13.6%)
Putting the medicines on the table at the beginning of a meal	11 (12.5%)

**Table 3:** Results from the Questions of Adherence Treatment Measure (MAT) and respective mean and median.

Question	Always %	Almost Always %	Often %	Sometimes %	Seldom %	Never %	Mean ± SD	Median
1. Have you ever forgotten to take the medicines for your illness?	0.00	0.00	2.3	18.2	47.7	31.8	5.09 ± 0.77	5.00
2. Have you ever been careless about the time you take your medicines?	0.00	0.00	5.7	22.7	31.8	39.8	5.06 ± 0.93	5.00
3. Have you ever stopped taking medicines for your illness because you felt better?	0.00	0.00	1.1	4.5	11.4	83.0	5.76 ± 0.59	6.00
4. Have you ever stopped taking the medicines for your illness on your own after feeling worse?	0.00	0.00	1.1	3.4	10.2	85.2	5.80 ± 0.55	6.00
5. Have you ever taken one or more pills for your illness on your own after feeling worse?	0.00	0.00	0.00	11.4	18.2	70.5	5.59 ± 0,69	6.00
6. Have you ever interrupted therapy for your illness because you have run out of medicines?	0.00	0.00	0.00	14.8	19.3	65.9	5.51 ± 0.74	6.00
7. Have you ever stopped taking your medicines for some reason other than a doctor’s appointment?	0.00	0.00	3.4	8.0	14.8	73.9	5.59 ± 0.78	6.00

**Table 4:** Minimum, maximum, mean, and median of the medication adherence levels.

**Adherence**	**Minimum**	**Maximum**	**Mean ± SD**	**Median**
	4.00	6.00	5.49 ± 0.40	5.57

**Table 5:** Patient-related problems according to PCNE.

Patient-Related Problems	*n* (%)
Patient intentionally uses/takes less drug than prescribed or does not take the drug at all for whatever reason	24 (27.7)
Patient unintentionally administers/uses the drug in the wrong way	4.0 (4.3)
Patient takes food that interacts	2 (2.2)
Patient stores drug inappropriately	1 (1.1)

## Data Availability

Data is contained within the article or supplementary material.

## Supplementary Materials

The following are available online at https://www.mdpi.com/article/10.3390/ijerph18147401/s1, Questionnaire, Table S1: Prescribed medicines according to ATC classification system.
