# Supplementary material for: Acceptance of Pharmaceutical Services by Home-Dwelling Older Patients: A Case Study in a Portuguese Community Pharmacy
Source: Int J Environ Res Public Health. 2021 Jul 11;18(14):7401. doi: 10.3390/ijerph18147401 (PMC8303520; doi:10.3390/ijerph18147401)
Supplement: Supplementary file 1 [file ijerph-18-07401-s001.zip › questionnaire_appendix 2.pdf]

Aplicação de um questionário sobre os problemas relacionados com medicamentos em idosos polimedicados utentes de uma farmácia na região da beira interior

Código do idoso: _____ Nº do questionário:___ Data da entrevista: __________

I -Rotina Diária e Organização dos medicamentos pelo doente idoso

(O idoso pode assinalar mais do que uma opção, se assim o desejar)

1. Qual é a perceção que tem da sua saúde?

Muito Boa __ Boa __ Média __ Má __ Muito má __

2. Quais os medicamentos que é mais frequente esquecer-se de tomar?

I. Os que toma em jejum. __
II. Os que toma ao pequeno-almoço. __
III. Os que toma ao almoço. __
IV. Os que toma ao lanche. __
V. Os que toma ao jantar. __
VI. Os que toma ao deitar. __
VII. NS/NR. __

3. Como identifica os seus medicamentos?

I. Pelo nome. __
II. Pela cor dos comprimidos/cápsulas. __
III. Pela caixa. __
IV. Pela forma dos comprimidos/cápsulas. __
V. Não os identifica, alguém os coloca numa caixa dispensadora de medicamentos. __
VI. NS/NR. __

4. **Na sua rotina diária por vezes sente dificuldades em:**

    I. Abrir os frascos dos medicamentos/blisters. __
    II. Engolir alguns medicamentos. __
    III. Recordar a forma correta de tomar alguns medicamentos. __
    IV. Gerir os horários de tantos medicamentos. __
    V. Utilizar alguns medicamentos (ex.inaladores). __
    VI. Não tem qualquer dificuldade. __
    VII. NS/NR. __

5. **Que estratégias utiliza para não se esquecer de tomar os seus medicamentos?**

    I. Coloca um alarme. __
    II. Utiliza uma caixa dispensadora onde coloca os do dia. __
    III. Utiliza uma caixa dispensadora onde coloca os da semana. __
    IV. Coloca as embalagens em sacos/locais separados, de acordo com o horário da toma (por exemplo os de almoço coloca todos juntos). __
    V. No início de cada refeição coloca na mesa os que tem de tomar. __
    VI. Outra. __ Qual?____
    VII. Nenhuma. __

6. **Quando o médico lhe prescreve um medicamento novo, costuma aconselhar-se com alguém antes de iniciar a toma?**

    Sim __ Não __ Se sim, com quem? _______________________________

7. **Com que frequência marca uma consulta de rotina no médico/a de família?**

    Semanal __ Mensal __ Trimestral __ Semestral __ Anual __ Outro ___________

8. **No último ano foi hospitalizado/a alguma vez?**

    Sim __ Não __ NS/NR __

8.1. Se sim, quantas vezes e porquê?_________________________________

8.1.1. Após a alta, houve alteração da medicação crónica antes do internamento?____

9. **No último ano foi às urgências alguma vez?**

Sim __ Não __ NS/NR __ Se sim, quantas vezes e porquê?_______________

II-Avaliação de Adesão à terapêutica (Medida de Adesão aos Tratamentos de *Delgado et al.)*

(Das seguintes opções de resposta o doente idoso deverá selecionar uma)

	**Sempre**	**Quase sempre**	**Com frequência**	**Por vezes**	**Raramente**	**Nunca**
**1.Alguma vez se esqueceu de tomar os medicamentos para a sua doença?**						
**2.Alguma vez foi descuidado com as horas da toma dos medicamentos para a sua doença?**						
**3.Alguma vez deixou de tomar os medicamentos para a sua doença por se ter sentido melhor?**						
**4.Alguma vez deixou de tomar os medicamentos para a sua doença por se ter sentido pior?**						
**5.Alguma vez tomou um ou vários comprimidos para a sua doença, por sua iniciativa, após se se ter sentido pior?**						
**6.Alguma vez interrompeu a terapêutica para a sua doença por ter deixado acabar os medicamentos?**						
**7.Alguma vez deixou de tomar os medicamentos para a sua doença por alguma outra razão que não seja a indicação do medico?**						

III- **<u>Conhecimentos dos idosos em relação aos seus medicamentos e identificação de algumas causas de problemas relacionados com os medicamentos</u>**

Número total de medicamentos que o idoso toma :_______________________________

Nº1	Nome comercial	
	**Forma farmacêutica e dosagem**	
	**Para que toma este medicamento?**	
	**Quantas vezes toma por dia? (C 7.7)**	
	**Quando toma ? (C 7.7)**	Jejum __ Pequeno almoço __ Almoço __ Lanche __<br>Jantar __ Ao deitar __ Não se lembra __
	**Há quanto tempo toma?**	Menos de 1 mês __Há mais de 1 mês e menos de 6 meses __<br>Mais de 6 meses__ Outro __ Não se lembra __
	**Tem que cortar o medicamento?**	Sim __ Não __ Se sim , porquê?
	**Alguma vez reduziu a quantidade deste comparativamente ao prescrito ou não tomou o medicamento? (C 7.1)**	Sim __ Não __ Se sim , porquê?
	**Alguma vez aumentou a quantidade deste medicamento mais do que aquilo que foi prescrito? (C 7.2)**	Sim __ Não __ Se sim , porquê?
	**Costuma tomar este medicamento com as refeições? (C 7.5)**	Sim __ Não __
	**Quando está fora de casa o dia todo, como faz para tomar este medicamento?**	Leva a medicação consigo __<br>Não toma nesse dia __<br>Toma quando chega a casa __<br>Leva consigo apenas os que acha mais importantes __<br>Toma antes de sair de casa __
	**Toma o medicamento com que bebida?**	
	**Onde costuma guardar este medicamento? (C 7.6)**	
	**Tem alguma dificuldade em tomar o medicamento? (C 7.9)**	Sim __ Não __ Se sim , qual?
	**Percebe as instruções que lhe foram dadas para tomar esta medicação? (C 7.10)**	Sim __ Não __ Se não , qual?

*** a tabela será repetida consoante o número de medicamentos que o idoso toma.**

1. **De todos os medicamentos, existe algum que considera mais importante? Qual/ais e porquê?**

    ____

2. **De todos os medicamentos existe algum que considera menos importante? Qual/ais e porquê?**

    ____

3. **Gostaria de ter alguém que a ajudasse com a gestão diária dos seus medicamentos?**

    Sim __ Não __ Se sim, porquê?_________________________________

IV-Serviços na farmácia (serviços esses que poderiam ser mais tarde aplicados caso os idosos adiram)

1.Estaria disposto a pagar para na farmácia haver um serviço de revisão da terapêutica que pudesse resultar num serviço mais avançado de seguimento farmacoterapêutico?

Sim __ Não __ Se não, porquê____

2.Estaria disposto a pagar para na farmácia haver um serviço de preparação individualizada de medicação para que tivesse a sua medicação devidamente separada e adequada para cada altura do dia em que tem de tomar?

Sim __ Não __ Se não, porquê?____

V-Caracterização Sociodemográfica do idoso

1.Sexo:

Feminino __ Masculino __ NS/NR __

2.Idade:

____

3.Estado civil

Solteiro(a) __ União de facto __ Casado(a) __ Viúvo(a) __ Divorciado(a) __

4. Com quem vive?

Sozinho (a) __ Cônjuge __ Outros ________________ NS/NR __

5.Que estudos completou?

Não sabe ler nem escrever__ Sabe ler e escrever sem concluir o grau de ensino __ 1ºciclo __ 2ºCiclo __ 3º Ciclo __ Ensino Secundário __ Ensino médio __ Ensino Superior __ NS/NR __

6.Os rendimentos que aufere encontram-se entre que limites?

<439€ __ 440-580€ __ 581-1160€ __ >1161€ __ NS/NR __

Tempo dispendido:___________________ A entrevistadora:_____________________

Medicação crónica do utente

(listagem de medicação inserida no SIFARMA)

1. ____
2. ____
3. ____
4. ____
5. ____
6. ____
7. ____
8. ____
9. ____
10. ____
11. ____
12. ____
13. ____
14. ____
15. ____
