# Supplementary material for: Acceptance of Pharmaceutical Services by Home-Dwelling Older Patients: A Case Study in a Portuguese Community Pharmacy
Source: Int J Environ Res Public Health. 2021 Jul 11;18(14):7401. doi: 10.3390/ijerph18147401 (PMC8303520; doi:10.3390/ijerph18147401)
Supplement: Supplementary file 1 [file ijerph-18-07401-s001.zip › supplementary material.pdf]

Table S1- Prescribed medicines according to ATC classification system

Prescribed medicine	Total number of consumed medicines	Number of patients (%)
A- Alimentary tract and metabolism	154	76 (86.4)
A02- Drugs for acid related disorders	58	57 (64.8)
A02B- Drugs for peptic ulcer and gastro-oesophageal reflux disease (gord)	58	57 (64.8)
A02BC- Proton pump inhibitors	55	55 (62.5)
A02BX- Other drugs for peptic ulcer and gastro-oesophageal reflux disease (gord)	3	3 (3.4)
A03- Drugs for functional gastrointestinal disorders	5	5 (5.7)
A03A- Drugs for functional gastrointestinal disorders	4	4 (4.5)
A03AB- Synthetic anticholinergics, quaternary ammonium compounds	1	1 (1.1)
A03AX- Other drugs for functional gastrointestinal disorders	3	3 (3.4)
A03F- Propulsives	1	1 (1.1)
A03FA- Propulsives	1	1 (1.1)
A05- Bile and liver therapy	3	3 (3.4)
A05A Bile therapy	3	3 (3.4)
A05AA- Bile acids and derivatives	2	2 (2.3)
A06- Drugs for constipation	4	4 (4.5)
A06A- Drugs for constipation	2	2 (2.2)
A06AC- Bulk-forming laxatives	1	1 (1.1)
A06AD- Osmotically acting laxatives	1	1 (1.1)
A09- Digestives, incl. Enzymes	2	2 (2.3)
A09A- Digestives, incl. Enzymes	2	2 (2.3)
A09AA- Enzyme preparations	2	2 (2.3)
A10- Drugs used in diabetes	68	43 (48.9)
A10A- Insulins and analogues	7	5 (5.7)
A10AB- Insulins and analogues for injection, fast-acting	1	1 (1.1)
A10AD- Insulins and analogues for injection, intermediate- or long-acting combined with fast-acting	2	1 (1.1)
A10AE- insulins and analogues for injection, long-acting	4	4 (4.5)

A10B- Blood glucose lowering drugs, excl. Insulins	61	41 (46.6)
A10BA- Biguanides	24	24 (27.3)
A10BB- Sulfonylureas	9	9 (10.2)
A10BD- Combinations of oral blood glucose lowering drugs	13	13 (14.8)
A10BG- Thiazolidinediones	1	1 (1.1)
A10BH- Dipeptidyl peptidase 4 (dpp-4) inhibitors	8	7 (7.9)
A10BK- Sodium-glucose co-transporter 2 (sglt2) inhibitors	6	6 (6.8)
A11- Vitamins	6	6 (6.8)
A11C- Vitamin A and D, incl. Combinations of the two	6	6 (6.8)
A11CC- Vitamin D and analogues	6	6 (6.8)
A12- Mineral supplements	8	8 (9.1)
A12A- Calcium	8	8 (9.1)
A12AX- Calcium, combinations with vitamin D and/or other drugs	8	30 (34.1)
B- Blood and blood forming organs	31	28 (31.8)
B01- Antithrombotic agents	29	28 (31.8)
B01A- Antithrombotic agents	29	2 (2.3)
B01AA- Vitamin K antagonists	2	1 (1.1)
B01AC- Platelet aggregation inhibitors excl. Heparin	18	17 (19.2)
B01AE- Direct thrombin inhibitors	1	1 (1.1)
B01AF- Direct factor xa inhibitors	8	8 (9.1)
B03- Antianemic preparations	2	2 (2.3)
B03A- Iron preparations	1	1 (1.1)
B03AA- Iron bivalent, oral preparations	1	1 (1.1)
B03B- Vitamin B12 and folic acid	1	1 (1.1)
B03BB- Folic acid and derivatives	1	1 (1.1)
C- cardiovascular system	223	86 (96.6)
C01- Cardiac therapy	18	17 (19.3)
C01A- Cardiac glycosides	8	8 (9.1)
C01AA- Digitalis glycosides	8	8 (9.1)
C01B- Antiarrhythmics, class I and III	1	1 (1.1)
C01BD- Antiarrhythmics, class III	1	1 (1.1)
C01D- Vasodilators used in cardiac diseases	5	5 (5.7)
C01DA- Organic nitrates	5	5 (5.7)

C01E- Other cardiac preparations	4	3 (3.4)
C01EB- Other cardiac preparations	4	3 (3.4)
C03- Diuretics	35	28 (31.9)
C03B- Low-ceiling diuretics, excl. Thiazides	12	12 (13.6)
C03BA- Sulfonamides, plain	12	12 (13.6)
C03C- High-ceiling diuretics	16	16 (18.2)
C03CA- Sulfonamides, plain	16	16 (18.2)
C03D- Potassium-sparing agents 7	7	7 (8.0)
C03DA- Aldosterone antagonists	7	7 (8.0)
C04- Peripheral vasodilators	4	4 (4.5)
C04A- Peripheral vasodilators	4	4 (4.5)
C04AD- Purine derivatives	4	4 (4.5)
C05- Vasoprotectives	7	7 (8.0)
C05C- Capillary stabilizing agents	7	7 (8.0)
C05CA- Bioflavonoids	6	6 (6.8)
C05CX- Other capillary stabilizing agents	1	1 (1.1)
C07- Beta blocking agents	19	19 (21.6)
C07A- Beta blocking agents	18	18 (20.5)
C07AA- Beta blocking agents, non-selective	1	1 (1.1)
C07AB- Beta blocking agents, selective	13	13 (14.8)
C07AG- Alpha and beta blocking agents	4	4 (4.5)
C07B- Beta blocking agents and thiazides	1	1 (1.1)
C07BB- Beta blocking agents, selective, and thiazides	1	1 (1.1)
C08- Calcium channel blockers	18	17 (19.2)
C08C- Selective calcium channel blockers with mainly vascular effects	15	14 (15.9)
C08CA- Dihydropyridine derivatives	15	14 (15.9)
C08D- Selective calcium channel blockers with direct cardiac effects	2	2 (2.3)
C08DB- Benzothiazepine derivatives	2	2 (2.3)
C09- Agents acting on the renin-angiotensin system	62	62 (70.5)
C09A- ACE inhibitors, plain	12	12 (13.6)
C09AA- ACE inhibitors, plain	12	12 (13.6)
C09B- ACE inhibitors, combinations	7	7 8
C09BA- ACE inhibitors and diuretics	3	3 (3.4)

C09BB- ACE inhibitors and calcium channel blockers	3	3 (3.4)
C09C- Angiotensin II receptor blockers (ARBS), plain	17	17 (19.3)
C09CA- Angiotensin II receptor blockers (ARBs), plain	17	17 (19.3)
C09D- Angiotensin ii receptor blockers (arbs), combinations	26	26 (29.5)
C09DA- Angiotensin II receptor blockers (ARBs) and diuretics	11	11 (12.5)
C09DB- Angiotensin II receptor blockers (ARBs) and calcium channel blockers	13	13 (14.8)
C09DX- Angiotensin II receptor blockers (ARBs), other combinations	1	1 (1.1)
C10- Lipid modifying agents	59	54 (61.1)
C10A- Lipid modifying agents, plain	56	51 (68.0)
C10AA- HMG CoA reductase inhibitors	43	43 (48.9)
C10AB- Fibrates	12	12 (13.6)
C10AX- Other lipid modifying agents	1	1 (1.1)
C10B- Lipid modifying agents, combinations	3	3 (3.4)
C10BA- HMG CoA reductase inhibitors in combination with other lipid modifying agents	3	3 (3.4)
D- Dermatologicals	1	1 (1.1)
D02-Emollients and protectives	1	1 (1.1)
D02A-Emollients and protectives	1	1 (1.1)
D02AX- Other Emollients and protectives	1	1 (1.1)
G- Genito urinary system and sex hormones	20	17 (19.3)
G03- Sex hormones and modulators of the genital system	5	5 (5.7)
G03C- Estrogens	4	4 (4.5)
G03CA- Natural and semisynthetic estrogens, plain	4	4 (4.5)
G03H- Antiandrogens	1	1 (1.1)
G03HA- Antiandrogens, plain	1	1 (1.1)
G04- Urologicals	15	13 (14.8)
G04B Urologicals	3	3 (3.4)
G04BD Drugs for urinary frequency and incontinence	2	2 (2.3)
G04C Drugs used in benign prostatic hypertrophy	12	10 (11.4)
G04CA Alpha-adrenoreceptor antagonists	9	9 (10,2)
G04CB Testosterone-5-alpha reductase inhibitors	2	2 (2.3)

G04CX Other drugs used in benign prostatic hypertrophy	1	1 (1.1)
H- Systemic hormonal preparations, excl. Sex hormones and insulins	(22)	22 (25.0)
H03- Thyroid therapy	(16)	16 (18.2)
H03A- Thyroid preparations	(14)	14 (15.9)
H03AA- Thyroid hormones	(14)	14 (15.9)
H03B- Antithyroid preparations	2	2 (2.3)
H03BA- Thiouracils	1	1 (1.1)
H03BB- Sulfur-containing imidazole derivatives	()	1 (1.1)
J- Antiinfectives for systemic use	2	2 (2.3)
J07- Vaccines	1	1 (1.1)
J07A Bacterial vaccines	1	1 (1.1)
J07AX- Other bacterial vaccines	1	1 (1.1)
L- Antineoplastic and immunomodulating agents	3	3 (3.4)
L01- Antineoplastic agents	1	1 (1.1)
L01B- Antimetabolites	1	1 (1.1)
L01BA- Folic acid analogues	1	1 (1.1)
L02- Endocrine therapy	2	2 (2.3)
L02B- Hormone antagonists and related agents	2	2 (2.3)
L02BG- Aromatase inhibitors	2	2 (2.3)
M- Musculo-skeletal system	(37)	29 (32.9)
M01- Antiinflammatory and antirheumatic products	20	17 (19.3)
M01A- Antiinflammatory and antirheumatic products, non-steroids	20	17 (19.3)
M01AB- Acetic acid derivatives and related substances	8	8 (9.1)
M01AE- Propionic acid derivatives	4	4 (4.5)
M01AH- Coxibs	3	3 (3.4)
M01AX- Other antiinflammatory and antirheumatic agents, non-steroids	4	4 (4.5)
M03- Muscle relaxants	4	4 (4.5)
M03B- Muscle relaxants, centrally acting agents	4	4 (4.5)
M03BX- Other centrally acting agents	4	4 (4.5)
M04- Antigout preparations	7	7 (8.0)
M04A- Antigout preparations	7	7 (8.0)

M04AA- Preparations inhibiting uric acid production	5	5 (5.7)
M04AC- Preparations with no effect on uric acid metabolism	2	2 (2.3)
N- Nervous system	(107)	58 (55.9)
N02- Analgesics	(14)	14 (15.9)
N02A- Opioids	7	7 (8.0)
N02AJ- Opioids in combination with non-opioid analgesics	6	6 (6.8)
N02AX- Other opioids	1	1 (1.1)
N02B- Other analgesics and antipyretics	7	7 (8.0)
N02BB- Pyrazolones	1	1 (1.1)
N02BE- Anilides	6	6 (6.8)
N03- Antiepileptics	12	12 (13.6)
N03A- Antiepileptics	12	12 (13.6)
N03AA- Barbiturates and derivatives	2	2 (2.3)
N03AE- Benzodiazepine derivatives	1	1 (1.1)
N03AG- Fatty acid derivatives	1	1 (1.1)
N03AX- Other antiepileptics	8	8 (9.1)
N04- Anti-Parkinson drugs	7	5 (5.6)
N04B- Dopaminergic agents	7	5 (5.6)
N04BA- Dopa and dopa derivatives	4	3 (3.4)
N04BC- Dopamine agonists	3	3 (3.4)
N05- Psycholeptics	(42)	32 (36.4)
N05A- Antipsychotics	7	5 (5.7)
N05AD- Butyrophenone derivatives	3	3 (3.4)
N05AH- Diazepines, oxazepines, thiazepines and oxepines	2	2 (2.3)
N05AX- Other antipsychotics	2	2 (2.3)
N05B- Anxiolytics	(34)	31 (35.2)
N05BA- Benzodiazepine derivatives	(33)	31 (35.2)
N05BB- Diphenylmethane derivatives	1	1 (1.1)
N05C- Hypnotics and sedatives	1	1 (1.1)
N05CF Benzodiazepine related drugs	1	1 (1.1)

N06- Psychoanaleptics	(28)	24 (27.2)
N06A- Antidepressants	(25)	23 (26.2)
N06AA- Non-selective monoamine reuptake inhibitors	1	1 (1.1)
N06AB- Selective serotonin reuptake inhibitors	13	13 (14.8)
N06AG -Monoamine oxidase A inhibitors	1	1 (1.1)
N06AX- Other antidepressants	11	11 (12.5)
N06B- Psychostimulants, agents used for ADHD and nootropics	1	1 (1.1)
N06BX- Other psychostimulants and nootropics	1	1 (1.1)
N06D- Anti-dementia drugs	2	2 (2.3)
N06DX- Other anti-dementia drugs	2	2 (2.3)
N07- Other nervous system drugs	4	4 (4.5)
N07C- Antivertigo preparations	4	4 (4.5)
N07CA- Antivertigo preparations	4	4 (4.5)
R- Respiratory system	(32)	14 (15.9)
R03- Drugs for obstructive airway diseases	(21)	14 (15.9)
R03A- Adrenergics, inhalants	15	13 (14.8)
R03AC- Selective beta-2-adrenoreceptor agonists	3	3 (3.4)
R03AK- Adrenergics in combination with corticosteroids or other drugs, excl. anticholinergics	11	11 (12.5)
R03B- Other drugs for obstructive airway diseases, inhalants	3	3 (3.3)
R03BA- Glucocorticoids	1	1 (1.1)
R03BB- Anticholinergics	2	2 (2.2)
R03D- Other systemic drugs for obstructive airway diseases	3	3 (3.3)
R03DA- Xanthines	1	1 (1.1)
R03DC- Leukotriene receptor antagonists	2	2 (2.2)
R05- Cough and cold preparations	1	1 (1.1)
R05C- Expectorants, excl. combinations with cough suppressants	1	1 (1.1)
R05CB- Mucolytics	1	1 (1.1)
R06- Antihistamines for systemic use	1	1 (1.1)
R06A- Antihistamines for systemic use	5	5 (5.7)
R06AX- Antihistamines for systemic use	5	5 (5.7)
S- Sensory organs	8	4 (4.6)

S01- Ophthalmologicals	7	3 (3.4)
S01E- Antiglaucoma preparations and miotics	7	3 (3.4)
S01EA- Sympathomimetics in glaucoma therapy	2	2 (2.3)
S01EC- Carbonic anhydrase inhibitors	2	2 (2.3)
S01ED- Beta blocking agents	2	2 (2.3)
S01EE- Prostaglandin analogues	1	1 (1.1)
S02- Otologicals	1	1 (1.1)
S02D- Other otologicals	1	1 (1.1)
S02DC- Indifferent preparations	1	1 (1.1)
